# The ethical permissibility of financial incentives

**DOI:** 10.1007/s11019-025-10315-1

**Published:** 2026-01-02

**Authors:** Pepijn Al, Jamie Brehaut, Katie Gillies, Justin Presseau, Mei-Lin Yee, Charles Weijer

**Affiliations:** 1 Department of Global Public Health and Bioethics, Julius center, UMC, Utrecht, The Netherlands; 2 Methodology and Implementation Research, Ottawa Hospital Research Institute, Ottawa, ON Canada; 3 School of Epidemiology and Public Health, University of Ottawa, Ottawa, ON Canada; 4 Aberdeen Centre for Evaluation (ACE), University of Aberdeen, Aberdeen, UK; 5 Patient partner, Montreal, Quebec, Canada; 6 Departments of Medicine, Epidemiology & Biostatistics, and Philosophy, Western University, London, ON Canada

**Keywords:** Recruitment, Financial incentives, Exploitation, Undue inducement, Voluntariness, Justice, Informed consent, Research ethics, Payments

## Abstract

Randomized controlled trials are central to progress in medicine, but trialists commonly struggle to recruit the required number of participants. Offering financial incentives is one proposed solution, but financial incentives may be problematic when they become irresistible and, thus, undermine the autonomy of potential participants. While some have dismissed this worry about undue inducement and argued that we should be paying participants more to avoid exploitation, we argue that this line of argument is based on a too narrow conception of undue inducement and does not fully appreciate the distinction between reimbursement, compensation, and financial incentives. On another, often overlooked concept of undue inducement, large incentives are an undue inducement when they are irresistible for people who do not want to participate and, thus, undermine voluntariness. This gives reason to limit the financial incentives to trial participants. We argue, furthermore, that large financial incentives could also be disproportionally attractive for people from economically marginalized groups and result in an unjust distribution of the burdens of research participation. We conclude that, while small sums of money are permissible as incentives, larger financial incentives should be avoided. We end by responding to the objection that keeping payments to participants small would be a form of exploitation. We argue that this argument does not apply to financial incentives. Unlike reimbursements and compensation, financial incentives are not payments to which participants can have a claim and, therefore, fall outside the scope of exploitation arguments.

## Introduction

Randomized controlled trials (RCTs) are central to the progress of medicine, but many trialists find it difficult to recruit enough participants (McDonald et al. 2006; Bower & Wilson, 2007; Bernardez-Pereira et al. 2014; Williams et al. 2015; Walters et al. 2017). A low recruitment rate can lead to the failure of a trial or the extension of the recruitment period. This exposes participants to unnecessary risks and wastes valuable resources that could be used for other trials (Kitterman et al. 2011; Williams et al. 2015). Low recruitment can also result in a smaller sample size, thereby reducing the statistical power of a trial (Altman 1980; Lenth 2001; Galea, 2007; Thoma et al. 2010). In the long term, recruitment failure can undermine medical progress. These difficulties have led trialists to search for ways to improve recruitment (Treweek et al. 2018a, b).

Recently, some authors have proposed to view trial recruitment through the lens of behavioral science by viewing the trial enrollment as a behavior (Brehaut, Lavin Venegas, et al. 2021; Gillies et al. 2021; Coffey et al. 2022). Trial recruitment, they argue, depends on multiple behaviors of different actors within the recruitment process (Gillies et al. 2021). All these behaviors, including trial enrollment itself, are affected by many factors. Whether someone participates in a study is, for example, heavily influenced by whether a physician talk to them about a trial and what recommendation the physician makes (Kinney et al. 1998). Driven by the multidisciplinary field of behavioral science, our understanding of what these factors are and how they influence behavior has grown immensely (Davis et al. 2015; Gillies et al. 2021). With this knowledge in hand, trialists can identify factors that influence the decision to participate and develop interventions that leverage those factors to increase recruitment.

One way to influence people’s behavior is to give them a financial incentive. This strategy has been taken up by some trialists, who have started to use financial incentives as a way to motivate people to participate in RCTs (Brown et al. 2012; Willard-Grace et al. 2013; Damschroder et al. 2014). There is increasing evidence that this use of incentives can have a positive effective on participation rates (Halpern et al. 2004, 2021; Cryder et al. 2010; Free et al. 2010; Jennings et al. 2015; Treweek et al. 2018a, b; Bickman et al. 2021; Abdelazeem et al. 2022). In a study conducted across 5 clinical trials, Jennings and colleagues found, for example, that offering £100 GBP in the invitation letter, which participants would receive after consenting to be screened, resulted in a 5% increase of consenting patients (Jennings et al. 2015).

Offering financial incentives—on top of reimbursements and compensation—to potential participants does, however, raise ethical questions. By offering potential participants a financial incentive, trialists attempt to influence the decision to participate of these people. While this is not problematic in and of itself, it may become problematic when the influence becomes irresistible. If that is the case, intentionally trying to influence the decision to participate by offering money becomes an controlling influence and, therefore, in tension with enabling people to make an autonomous decision about trial participation (Faden and Beauchamp 1986). Since it is important that this decision to participate is made autonomously, this is a reason to be hesitant with financial incentives.

A number of ethicists have argued that these worries about high payments are overblown (Wilkinson & Moore, 1997; Savulescu 2001; Emanuel 2004, 2005; Largent & Fernandez Lynch, 2017). They argue that payments to participants, including financial incentives, are acceptable when the risks of a trial are reasonable (Emanuel 2004, 2005; Largent & Fernandez Lynch, 2017). When a trial has passed ethics review, it will not impose unreasonable risks on participants and it would, therefore, be ethically permissible to offer money to people to persuade them to participate in that trial.^1^ Largent and Fernandez Lynch have even argued that “the default approach to payment should be flipped—rather than assuming payments are too high, [institutional review boards] should start asking whether payments are high enough (Largent & Fernandez Lynch, 2017, 1).” When payments are too low, they argue, trials may take advantage of their participants—thus, subject them to exploitation. So, according to this argumentation, high financial incentives are not only ethically permissible, they are ethically required.

These discussions of the ethics of payments to participants have, however, two notable shortcomings. Firstly, they have mainly focused on a specific interpretation of “undue inducement”: a large payment that induces people to take a risk that they should not or would not take in absence of that payment (Emanuel 2004, 2005; Appelbaum et al. 2009; Nelson et al. 2011; CIOMS 2016; Largent & Fernandez Lynch, 2017; SACHRP 2019; Gelinas 2020; Gelinas et al. 2020). As a result, they have largely overlooked the ethical worry that large payments may undermine voluntariness, even if they do not result in increased risk taking. Secondly, the exploitation argument does not fully appreciate the distinction between different forms of payment, i.e., between reimbursement, compensation, and financial incentives. While small payments could indeed constitute a form of exploitation for compensation, we argue that this argument does not apply to financial incentives. Both these points impact the ethical evaluation of financial incentives.

In this paper, we will give an ethical evaluation of financial incentives that takes these points into account. Based on another conception of undue inducement and the ethical concern that large incentives can result in an unjust distribution of the burdens of trial participation, we will argue that small monetary amounts of incentives to increase participation rates among healthy participants and patient-participants are permissible, but that large incentives are impermissible.^2^ Although financial incentives do not result in more risk taking among potential participants and the risk of deception that they might motivate can be mitigated, there are other reasons to limit them. Financial incentives can diminish the voluntariness of people that decide to participate in a trial and contribute to an unjust distribution of burdens. While incentives can have benefits, these benefits are outweighed by concerns about voluntariness and justice when the incentives are large. It would, therefore, be preferable to restrict financial incentives to small sums of money. As we will argue at the end of the paper, drawing an exact line will be difficult and requires more empirical research, but financial incentives should generally be small and should not exceed an amount that provides reason to believe that the incentive imperils voluntary consent. In the absence of more empirical data, we suggest setting this limit for financial incentives of 100 euros, though more research needs to be done to further specify and justify such a limit.

This paper has the following structure. We will first discuss what financial incentives are by distinguishing them from reimbursement and compensation to clarify the focus of this paper. We will then consider three forms of the objection that financial incentives are an “undue inducement”. While the first two forms of undue inducements do not offer strong reasons for limiting incentives, we argue that the final, often overlook form of undue inducement does. Next, we will argue that financial incentives can result in an unjust distribution of the burdens of research. Based on this discussion, we conclude that financial incentives to research participants should be kept small. We end by refuting an argument that is commonly made in debates about the permissibility of payments to participants: that keeping payments to trial participants small is a form of exploitation. This argument, we will argue, does not apply to financial incentives as financial incentives are not given in return for any assumed burdens.

## What are financial incentives?

There are three types of payments that research participants may receive when participating in a trial: reimbursement, compensation, and incentives (Dickert & Grady, 1999; Largent et al. 2012; Gelinas et al. 2020; Bierer et al. 2021).^3^ These forms of payment can be distinguished based on their justification, though they are not mutually exclusive. Reimbursements are meant to cover out-of-pocket expenses of participants when they participate in a trial, such as travel or parking costs, and are, thus, conditional on costs incurred. They are justified based on the idea that “participants should not have to pay for making a contribution to the social good of research (CIOMS 2016).” Compensation is meant to make up for participants’ time spent as research participants and the inconvenience of research participation and is, thus, justified by the time spend in a trial and burdens of trial participation. Financial incentives, in contrast to compensation and reimbursement, are payments that are intended to motivate a person to participate in a study without appeal to the costs incurred or the time spend as a research participant.^4^ They are justified by the need for trial participants for socially valuable research and not based on the time spend in a study or costs that a participant makes. Their sole purpose is to motivate people to participate in research. Although other forms of payment can also do this, incentives are payments that go “beyond what would be justified for reimbursement and compensation (Gelinas et al. 2018, p. 770).”

While this distinction is often used in ethical discussions, it is not uncontroversial, as these different types of payments cannot always be neatly differentiated. Compensation can, for example, also function as an incentive. According to Różyńska this problem arises because this categorization mixes different criteria (Różyńska 2022). Reimbursement and compensation can be identified based on their effect on a participants economic position: reimbursements ensures that no costs are incurred and compensation gives additional payment to the participants in exchange for the time and burdens.^5^ Financial incentives, in contrast, are based on the intention of the researcher. The different types of payment are, furthermore, often not distinguished in practice; guidelines, legal documents, and protocols rarely distinguish incentives as a separate category (CIOMS 2016; SACHRP 2019; WMA General Assembly 2024). In other cases, incentives are taken to be synonymous with payments for participation (FDA 2018). This also explains the lack of empirical data about how often financial incentives are given in practice.

While we acknowledge that this distinction can get blurry, we think it is possible and important to uphold it. Firstly, while intentions and economic outcomes are important for the different categories, the central question for the categorization is: how these payments are justified? Answering this question does not only make it possible to separate different payments, but also gives a reason for doing so; since the different types of payment have different justifications, they raise different considerations and objections. These considerations and objections can best be examined when financial incentives are discussed separately.

Secondly, there are good reasons to also make this distinction in practice. Distinguishing reimbursements, compensation, and incentives in guidelines and policy documents acknowledges the different ethical considerations of these types of payment and gives trialists more specific guidance on what they can offer to participants. While some guidance documents already distinguish reimbursements and compensation, incentives are often not addressed separately or not at all (CIOMS 2016; SACHRP 2019; FDA 2018; WMA General Assembly. 2024). Discussing incentives separately will give both trialists and ethics committees more guidance about what can be offered on top of reimbursement and compensation. Making the same distinction in protocols and informed consent documents will also give transparency to trial participants about why they are paid and why this amount.

Thirdly, financial incentives seem to be more controversial than the other types of payments. In the most comprehensive survey on this subject to date, 97% of 610 research ethics professionals in the United States said that reimbursing healthy volunteers was ethically acceptable, while only 58% of respondents thought that it was ethically acceptable to motivate healthy volunteers to participate through financial incentives (Largent et al. 2012).^6^ Similar attitudes were expressed by trial participants of a vaccine trial conducted in Southern Alberta (Russell et al. 2000). Altogether, this gives reason to discuss the ethical permissibility of financial incentives separately.

The focus of this paper is on the ethical permissibility of *financial* incentives that are *conditional* on study enrollment as opposed to *non-monetary* incentives and *unconditional* incentives.^7^ We will also discuss the ethical permissibility of financial incentives to patient-participants and healthy volunteers largely together. We will distinguish the different considerations related to healthy volunteers and patient-participants when relevant—specifically, we will discuss considerations about justice in light of the different demographics of these groups—but we do not think the differences between healthy volunteers and patient-participants require a totally separate ethical analysis. While patient-participants might, for example, be less motivated by money than healthy volunteers (Stunkel and Grady 2011; Moorcraft et al. 2016; Fisher 2020; Brehaut et al. 2021a), this population is diverse and will include people that would be sensitive to financial incentives. Thus, if our argument is correct, large financial incentives can constitute an undue inducement for both patient-participants and healthy volunteers. 

## Undue inducement

The debate on the ethical permissibility of financial incentives centers around different conceptions of undue inducement—sometimes also referred to as “undue influence”. Undue inducement is used to refer to multiple, problematic influences that offers can have on potential participants. One form of undue inducement is when large incentives alter potential participants’ perception of the risks involved in participating, thereby undermining consent and causing people to take on risks that they should not, or would not otherwise take (McNeill 1997; CIOMS 2016). Secondly, undue inducement sometimes refers to inducements that motivate ineligible people to conceal important information that would exclude them from participating (Macklin 1981). As a result, these participants could be exposed to high risks caused by the characteristics that made them ineligible. Finally, large financial incentive could also be an undue inducement when they become irresistible for people who would otherwise not participate. This can result in diminished voluntariness in the decision to participate.

While the first two forms of undue inducement have received ample attention in the literature, the last form has been largely overlooked. When concerns about voluntariness are discussed, the concern often boils down again to concerns about risk perception. In this section, we will discuss these different conceptions of undue inducement in turn and argue that this focus is unjustified; while critics are correct in dismissing risk perception and deception as reasons to limit financial incentives, but voluntariness is a basis to limit financial incentives.

### Risk perception

Financial incentives could be an undue inducement when they are so large that people do not accurately perceive the risks of a trial and, thus, undermines their ability to make a good judgement about participation (CIOMS 2016; Appelbaum et al. 2009). This could give rise to two problems. Firstly, this form of undue inducement could invalidate consent, as consent would not be based on an adequate understanding of the trial, leading them to consent to risks that they otherwise would not take (Appelbaum et al. 2009; SACHRP 2019; Gelinas 2020; Gelinas et al. 2020). Secondly, an altered perception of risks could lead people to consent to participate in a trial even though it is contrary to their interests (Emanuel 2004, 2005; Nelson et al. 2011; Largent & Fernandez Lynch, 2017).

This worry has been widely discussed and there are good reasons to dismiss it. Firstly, empirical research suggests that large incentives do not alter people’s perception of the risks of a trial (Halpern et al. 2004, 2021; Bickman et al. 2021). To the contrary, large payments may make people more cautious of potential risks (Cryder et al. 2010). Secondly, even if people tend to take more risks when they are offered money, these risks are not unreasonable for most people. As Emanuel pointed out, while trial participation always involves risks, there are safeguards to predict and minimize these risks (Emanuel 2004, 2005). These ethics safeguards could fail, but this is, as Emanuel argues, a failure of the system that is supposed to protect research participants, not a form of undue inducement (Emanuel 2004).

### Deception

While the typical participant will not be exposed to excessive risks when agreeing to participate in a trial that passed ethics review, there are specific groups for whom participating in a trial may still be excessively risky. These groups are excluded from the trial through eligibility criteria, but large financial incentives can motivate those people to conceal important medical information to remain eligible for participation. In that case, incentives can also be called “undue inducement” (Macklin 1981). This worry is supported by empirical evidence, which shows that financial incentives can and does indeed motivate potential participants to deceive trialists (Bentley and Thacker 2004; Devine et al. 2013; Fernandez Lynch, 2019). In the face of large financial benefit, participants could, for example, conceal information about their medical history, the drugs they are taking, or their past participation in other trial (Devine et al. 2013). Such deception can endanger the safety of participants and the scientific integrity of trials.

It is, however, uncertain whether keeping financial incentives small would minimize the risk of deception. Since money can be an important motivation for deception, it is plausible that smaller financial incentives would also reduce deception among participants. The research of Fernandez Lynch and colleagues suggests, however, that larger payments do not increase deception (Fernandez Lynch et al. 2019). More empirical research is needed to settle this question. Moreover, safeguards—such as objectively verifiable eligibility criteria and a database or registry for research participants (Resnik & Koski, 2011; Pavletic, 2017)—can and should be implemented to minimize the possibility for deception (Resnik and McCann 2015; Largent & Fernandez Lynch, 2017; Gelinas et al. 2018). In the presence of adequate safeguards, the use of modest financial incentives is ethically acceptable.

### Voluntariness

Substantial financial incentives can also be a form of undue inducement when they diminish a participant’s voluntariness. While empirical research is lacking, there is reason to be concerned about the effect of incentives on voluntariness. Qualitative research shows that money can be a strong motivation for people to participate in a trial, especially for healthy volunteers (Stunkel and Grady 2011; Fisher 2020). This is further underlined by the positive effect that incentives can have on recruitment and people’s willingness to participate (Halpern et al. 2004, 2021; Cryder et al. 2010; Free et al. 2010; Jennings et al. 2015; Treweek et al. 2018a, b; Bickman et al. 2021; Abdelazeem et al. 2022). When a financial incentive is large enough, however, this motivating force can also sway people to participate in a trial, even if they have a preference against participating. The worry is that a person does not want to participate, but does reluctantly enroll in the trial because they need the money.

Being motivated by financial incentives does, of course, not always lead to involuntary participation. What we want, including whether we want to participate, is determined by multiple factors, of which financial incentives are only one. These factors can be ordered along a continuum from intrinsic to extrinsic reasons for acting (Ryan & Deci, 2002, 2020; Howard et al. 2017). Autonomous actions are typically more intrinsically motivated, while extrinsic reasons typically make an action less autonomous.^8^ But while financial incentives lay on the more extrinsic end of the continuum, they are compatible with being intrinsically motivated. Even when people are primarily motivated by extrinsic reasons, they can act voluntarily—many people go to work voluntarily for extrinsic reasons, i.e., money. Financial incentives can, thus, be a reasonable motivation to join a trial and not every person that participates in a trial because of a financial incentive does so involuntarily.

But while extrinsic reasons can be compatible with voluntariness, they can also result in involuntary actions. A financial incentive can lead to involuntary participation when it motivates a person, who has strong preferences against participating in a trial, to reluctantly participate. This is the case, for example, when a person has a strong reason against participating because they distrust the researchers or research institution, but enrolls because they find it hard to decline the financial incentive. This person ends up participating, because they found themselves unable to resist the financial incentive, even though they want to. It is this lack of resistibility that makes an incentive controlling (Faden & Beauchamp, 1986; Saghai 2013).^9^

This form of undue inducement is often overlooked in debates on payments to participants. Both Appelbaum and colleagues, and Nelson and colleagues acknowledge that large payments can be a form of (illegitimate) control, but they also require an extra condition for this to be problematic (Appelbaum et al. 2009; Nelson et al. 2011). For Nelson and colleagues this is “a risk of harm of sufficient seriousness that the person’s welfare interest is negatively affected by assuming it” (Nelson et al. 2011, p. 9). For Appelbaum and colleagues an offer is problematic when it is “substantial enough to undermine a person’s decision-making process—in other words, if it leads a person to ignore or undervalue the risks of research participation” (Appelbaum et al. 2009, p. 34).

The potential of high incentives to undermine voluntariness is, however, in itself an important ethical worry. Controlling influences are seen as incompatible with making an autonomous choice (Faden & Beauchamp, 1986). This is for a good reason: making a decision in the presence of substantially controlling influences can result in people participating against their will. This is ethically problematic independent of the risks of participating, as it violates the principle of respect for persons. This worry is overlooked when increased risk taking is a condition for rendering a financial incentive problematic.

Being motivated by money is, as said, not problematic on its own. Furthermore, some reluctance—e.g., “I would rather do something else if I had more options”—does not even have to be problematic either. So, at what point does this become problematic? Although drawing an exact line between problematic and unproblematic money-motivated participation is difficult, we think that the concept of authenticity, as developed by Christman, is helpful to take a first step in that direction (Christman 2009).^10^ What makes participating against your will problematic is not just that the participation is done with some reluctance or externally motivated, but that the decision to participate is no longer a person’s own but imposed on them by outside influences. In Christman’s account of authenticity, a person’s decision, commitment, or value is inauthentic when they would feel alienated from that decision, commitment, or value when they would reflect on it in light of the process that brought about the decision. Thus, if a person feels strongly alienated from their decision to participate that was made in light of the financial incentive offered, there is reason to see this decision as something that was imposed through external rather than internal influences, as a decision that is involuntary and not autonomous.^11^

Alienation from the decision to participate can be measured indirectly by measuring regret and dropout rates in trials. Although regretting a decision and dropping out of a trial does, of course, not necessarily imply alienation from the decision to participate, these are potentially good proxy measures. People who feel alienated from their decision to participate will more likely regret their decision and withdraw from a trial. Higher regret and dropout rates than is seen in comparable trials with smaller incentives can indicate that the decision to participate was not authentic. Measuring dropout rates is relatively easy to implement in trials, as dropout rates are already reported in CONSORT flow diagrams that are included in most trial reports (Altman et al. 2001). There are different instruments to measure regret, which could be assessed through surveys (Joseph-Williams et al. 2010). While this may require some extra work, it would be a good way to measure the effects of financial incentives when they are used.

Some have challenged the argument of undue inducement by comparing trial participation with (risky) work (Savulescu 2001; Emanuel 2004, 2005; Arnason and Van Niekerk 2009). Incentives to do things you would otherwise not do—e.g., take a job—are common and uncontroversial for work. So, why should we consider similar incentives unethical when offered in exchange for trial participation? This has, Emanuel argues, problematic implications: “Indeed, inducements are so commonplace and acceptable that our daily lives would be drastically different if they were all prohibited as unethical (Emanuel 2004, p. 100).”

There are two problems with this line of reasoning. Firstly, trial participation is not just another job (Różyńska 2018). While most work requires a kind of skill, this is not required for trial participation. Trial participation involves, in contrast to most jobs, letting others use your body to test and endure something. Even “‘model patients’, who are paid by medical schools for undergoing physical examinations performed by students”—a counterexample mentioned by Różyńska—require some type of skill, namely acting (Różyńska 2018, p. 639). While there might be other counterexamples, the fact that these examples are not obvious indicates that we should be cautious with drawing conclusions about the ethics of trial participation based on our intuitions about work.

Secondly, it is important to keep in mind that the standards of informed consent and voluntariness for research participation are higher than for most other activities. Even if we consider trial participation a job, it is a very peculiar one that warrants extra protections. RCTs intervene either on people’s bodies or otherwise on their lives for the benefit of society and, in many cases, for the direct benefit of participants. People have a special interest to decide what happens to their body (Dworkin 1988). To respect people’s bodily integrity, we have to make sure that participation in a trial is voluntary. Having strong protections to preserve a level of voluntariness also helps to maintain trust in medical research (O’Neill 2002). Trust in medical research and research institutions could be undermined if we allow people to be enrolled in RCTs involuntarily. While it might be acceptable that some people go to the office reluctantly, protecting bodily integrity and trust in medicine requires that the standards of voluntariness are higher for trial participation.

### Are undue inducements a reason to limit financial incentives?

We have considered three ways in which an inducement can be undue. The first type was the idea that inducements can be so large that they influence people’s risk perception in such a way that they undermine their ability to make good decisions. Empirical and philosophical arguments show both that this is not a legitimate concern in practice and, therefore, that this is not a reason to limit financial incentives. The second type of undue inducement was when an incentive is so large that it motivates people for whom participating in a trial may be excessively risky to conceal important medical information to remain eligible for participation. While this is a problem observed in practice, limiting financial incentives is neither necessary nor sufficient to overcome this. Finally, we have argued that there is a third type of undue inducement, which we should be worried about. This is when incentives are so large that they undermine the voluntariness of a person’s decision to participate. This type of undue inducement gives reason to limit financial incentives.

## Justice

In addition to worries about undue inducement, large sums of money could also lead to an unjust distribution of the burdens of research participation. For a just research practice, it is important that the burdens of research participation should not disproportionately be carried by one group (Emanuel et al. 2000; MacKay and Saylor 2020). Large sums of money could mainly incentivize poorer people to participate, while being less attractive for the rich. Assuming that the rich also stand to benefit from the trial, a situation where poor people disproportionally carry the burdens of research, while the rich would also benefit from research, would violate distributive justice and, thus, be unjust.

Proponents of large payments have argued that larger incentives will have the opposite effect: it would contribute to a more just distribution of the burdens of research (Wilkinson, 1999; Emanuel 2004, 2005; Largent & Fernandez Lynch, 2017). According to these authors, large payments make trial participation more appealing for people with greater pre-existing financial means. Larger incentives could also make trial participation more appealing for people from underserved communities (Ibrahim, 2014; Owusu-Addo, Ebenezer et al., 2024). If this is the case, we have reason to increase incentives to promote a more equitable and, thus, just distribution of the burdens of research over the population instead of keeping incentives low.

How the burdens of research participation are distributed is indicated by the demographic data. Because the demographics of patient-participants and healthy volunteers are different, it is useful to distinguish these groups here. We will start by considering the effect of higher incentives on the distribution of the burdens of research within patient-participants and then do the same for healthy volunteers. We argue that, while there is too little empirical research to draw any definitive conclusions about which hypothesis is correct, current data suggest that higher incentives will not lead to a more more equitable distribution of the burdens of trial participation. To the contrary, there is a legitimate worry that higher incentives will lead to a more unjust distribution of burdens.

While surveys suggests that patient-participants mainly consist of people with a higher income (Unger et al. 2013; Kim et al. 2024), it is questionable whether higher incentives can promote better representation of people from lower socioeconomic background in the patient-participant population. Halpern and colleagues found that larger payments had a stronger effect on the willingness to participate in a hypothetical trial of wealthier patients, not poorer patients (Halpern et al. 2004). Research done within the context of real trials neither supports the idea that higher incentives will lead to better representation. In a study embedded in five real-world trials, Jennings and colleagues found that incentives have the strongest effects on patients from middle-income groups (Jennings et al. 2015). In a later study, which was embedded in two real trials, Halpern and colleagues also found that there is no significant difference between the effect of payments on willingness to participate between patients from different income groups (Halpern et al. 2021). Thus, higher incentives do not seem to promote an equitable distribution of burdens in trials with patient-participants.

The demographics of healthy volunteers are different: studies on professional research participants indicate that these participants are mainly people from economically disadvantaged groups in real life (Elliott 2008; Fisher 2008, 2020; Abadie 2010; Fisher and Kalbaugh 2011). Thus, the burdens of trial participation mainly fall on poorer people. It may be argued that higher financial incentives could help here, as money is the main motivation of healthy volunteers. There is, however, no empirical research that supports this assumption. Giving higher incentives may incentivize wealthier participants, but it may also incentivize even more poorer people before being attractive for wealthier people. There is reason to believe that the second option is correct, as the current demographics of healthy volunteers suggest that incentives for trial participation with healthy volunteers primarily attracts people from a disadvantaged socioeconomic background. This would mean that the distribution of the burdens of research would even be more unjust. Because of the injustice of disproportionately placing burdens of research on the poor and the current demographics of professional trial participants, those who claim that larger payments will lead to a more equitable distribution of burdens among healthy volunteers have a burden of proof. This burden of proof is currently not met. More research needs to be done within existing trials and it is important that trialists track the use of incentives and the socioeconomic status of approached and enrolled participants. Without this research, we should err on the side of a caution and keep financial incentives small.

It may be argued that this choice for keeping incentives low accepts under-enrolment in trials in order to avoid an unequitable distribution of the burdens of research.^12^ While small incentives may contribute to a more equitable distribution of the burdens of research participation, it limits trialists options for overcoming the challenge to recruit. Low recruitment is, as discussed in the introduction, a structural problem that makes everybody worse off. Financial incentives are a potentially effective way of increasing participation that are relatively easily introduced in the recruitment process. A higher participation rate reduces the number of trials that fail or have to be extended. As a result, the number of people that are exposed to unnecessary risks due to a failed trial will decrease as well as the economic costs of a failed or extended trial. Another important benefit is that trails that would have failed without the financial incentives can generate scientific knowledge that contributes to medical progress. This can potentially improve people’s lives or even save them. So, if that is the case, why not offer high incentives? This may result in a more inequitable distribution of the burdens of research participation, but it could also a higher participation of wealthier people and contribute to better recruitment rates.

A rejection of large financial incentives does, however, not imply that we should just accept low participation rates or hope that people will become much more altruistic. It is, first of all, not clear that large incentives are indeed much more effective for recruiting than small incentives. A small incentive, such as £5 GBP, can already have a positive impact on recruitment (Free et al. 2010; Abdelazeem et al. 2022). Secondly, there might also be other ways to increase participation; incentives are only one of 93 different behavior change techniques (Michie et al. 2013). Even though money is often the primary motivation of healthy participants (Stunkel and Grady 2011; Fisher 2020), we should not opt for financial incentives as the main motivator of research participants. We should, instead, explore other, potentially more ethically acceptable, alternatives.

If limiting financial incentives does not fundamentally undermine trialists ability to increase trial participation, the choice comes down again on a question about justice: smaller incentives, risking a less equitable distribution of burdens because wealthier people are not motivated by these incentives; or larger incentives, risking a less equitable distribution of burdens because those larger incentives mainly attract poorer people. Given the current demographics of professional research participants, we think the first option is the better choice.

## Limiting financial incentives and exploitation

We have considered four arguments against the use of large financial incentives to increase participation rates—three forms of undue inducement and one argument based on concerns around a just distribution of burdens. While large incentives may not result in undue risk-taking and the risk of deception may be minimized by implementing safeguards in the recruitment process, the potential impact of large incentives on voluntariness and the just distribution of the burdens of participation give reasons to keep financial incentives small. Although financial incentives can also have important benefits for participant recruitment, these benefits should be balanced against these ethical problems with large incentives. All things considered, small financial incentives to trial participants are permissible, but we should not give large financial incentives to participants.

One common objection against putting a cap on payments to trial participants is that this would result in the exploitation of trial participants (Wilkinson and Moore 1997; Emanuel 2004; Largent & Fernandez Lynch, 2017; Gelinas et al. 2020). Exploitation is broadly defined as taking an unfair advantage of someone. In the context of research participation, it is argued that participants can be exploited if they are not paid or paid too little in exchange for the burdens they assume (Largent & Fernandez Lynch, 2017). This is especially problematic when researchers, institutions, and a society gain substantively from the research. There is, thus, an unfair distribution of benefits in relation to the burdens the different parties in the research practice assume. If this is correct, we should strive to pay trial participants more, not less, in order to avoid exploitation.

This argument does not, however, apply to financial incentives. Financial incentives are given either in addition to compensation and reimbursement, or when compensation and reimbursement is not necessary. They are, moreover, not based on assumed burdens but intended to motivate people. Financial incentives are, thus, not concerned with the fair distribution of benefits in relation to assumed burdens; they are solely intended to motivate people to participate in a trial. This does not mean that exploitation of trial participants cannot or does not occur, but this simply not the domain of financial incentives—avoiding exploitation is a concern for compensation.

This has two implications. Firstly, given that financial incentives are not depended on assumed burdens, no participant has a claim to an incentive. Offering no or only a small financial incentive can, thus, not amount to exploitation. Exploitation is, thus, not a valid objection against limiting financial incentives. Secondly, since financial incentives are not depended on burdens and benefits, whether a participant, often patient-participants, benefits from participating in a trial does not impact the question whether and what financial incentive is appropriate. For the same reason, not offering an incentive does not deprive people of an option, as some might suggest (Wilkinson, 1999; Grady 2001; Beauchamp et al., 2002). Since no one has a claim on a financial incentive, no one can be deprived of this incentive.

It will be difficult to draw an exact line between small, permissible financial incentives and larger impermissible incentives. On an abstract level, large incentives are those about which we have reason to believe that they imperil voluntary consent. As practical guidance, we suggest a limit of 100 euros. However, given that there is only limited empirical data on the effects of incentives, and that what are considered large incentives will vary among groups (Macklin 1981), it is important that we get more data on the effects of financial incentives in real trials and in different contexts to specify and justify a threshold. To get these insights, it is important that trialists that use financial incentives keep track of important data, such as the demographics of their participants and retention rates. Studies within a trial may be a particularly useful avenue to test the effects of financial incentives in comparison to no incentives (Treweek et al. 2018a, b). Questions of particular interests that could be addressed in studies within a trial are:

- Do the baseline characteristics, such as socio-economic background, change when large financial incentives are offered? And if so, how do they change?
- How many people dropout from the arm that offers financial incentives and how many participants regret participation? Are these dropout rates and number of participants with regret different in the arm without financial incentives and how do they differ? What is the effect on these numbers of larger financial incentives?^13^

To answer these questions, it may be permissible to give larger financial incentives in studies within a trial.

## Conclusion

In this paper, we have argued that, at this point, only small financial incentives are ethically permissible to increase participation rates. The use of big financial incentives is too much in tension with the principles of justice and respect for persons. The argumentation in this paper is limited by the lack of empirical data on the effects of financial incentives. In the absence of such data, there is reason to be cautious and to assume that financial incentives lead to involuntary participation and to an unjust distribution of the burdens of trial participation. If new empirical data would, however, disprove these assumptions, there is reason to re-evaluate the arguments in this paper.

## Footnotes

1 This is, of course, under the assumption that the people who to which money is offered are decisionally competent.

2 We will focus on the effects of financial incentives on the initial decision to participate only and not discuss financial incentives that are aimed to increase retention. It is likely, however, that financial incentives that are given for staying in a trial will raise similar concerns as those that are given upon participation, such as concerns about voluntariness. Although the discussion in this paper can inform a discussion of retention incentives, a separate discussion of those incentives is necessary.

3 Note that we only consider payments that people receive for participating in a trial. There are other ways in which people may be involved—patient representatives may, for example, be consulted about the design of a trial—for which they may receive an honorarium. Since such types of involvement fall outside the scope of this paper, we will neither consider those types of payments.

4 In behavioral science, a distinction can be made between informing potential participants that they *will* receive money when they enroll, which called an “incentive”, and giving money after a person enrolled, which is called a “reward”. So, incentives work by promising money—for example, by indicating the money that will be given to participants in consent documents—while rewards work by attaching value to desired behavior—for example, by providing a reward at different intervals for participating in different phases of data collection. Because we are interested here in the ethics of using a behavioral science technique to increase *recruitment*, not retention, we will focus on incentives and not on the rewards, as rewards are given after enrollment and, therefore, primarily encourage retention in a trial.

5 Różyńska’s distinction is actually a distinction between recompense and remuneration. Recompense includes payments that does not bring a net benefit to the participant, but makes sure that they do not incur any losses due to their participation in a trial. Remuneration is any payment that is intended to benefit a participant, for example in exchange for their contribution. We interpret this as mapping on our distinction between reimbursement and compensation, but it could be argued that compensation should be seen as a way of making up for the time spend as a research participant and, for example, cover lost wages. This would make it a form of recompense, not remuneration. Whether compensation is recompense or remuneration depends on the baseline of the participant—for a participant with no wages lost, compensation will be a form of renumeration. What is important, however, is that incentives cannot be described in relation to a baseline; they are not meant to cover losses compared to a baseline or to make a participant better off, they are solely intended to incentivize people to participate.

6 It might be that respondents to the survey had the definition of the FDA in mind when thinking about incentives, in which the tripart division is not used and all forms of payment to participants are considered recruitment incentives. While this is possible, it is important to note that Largent and colleagues also asked about reimbursements and compensation in their survey. While we do not how the questions were presented in the survey, this does suggest that the tripart division was presented to the respondents and that they responded differently to the different justifications for offering money to participants.

7 Unconditional incentives are given to induce a reaction of reciprocity on the part of potential participants (Cialdini 2001). Some surveys, for example, send a small sum of money (e.g., $40 USD, $1 USD, $0.10 USD) in advance to potential respondents to increase the response rate (Jobber and O’Reilly 1996; Laurie and Lynn 2009). This mechanism of inducing reciprocity is different than the mechanism of action of conditional financial incentives, which are not given to induce reciprocity but intended to make the option of participating more appealing.

8 Note that, although autonomous actions are *typically* primarily intrinsically motivated, being autonomous is not necessarily the same as acting on intrinsic reasons. Some extrinsic reasons may, for example, be internalized through oppressive socialization. As a result, a person may act on intrinsic reasons, but not autonomously.

9 Note that an influence can be problematic without being an irresistible threat—i.e., coercion. The influence of family members in treatment decisions may be a helpful analogy. While the advice or wishes of family members may be a legitimate reason to consent to a treatment, it can also be problematic when their wishes become too dominant in the decision (Blackler 2016; Laidsaar-Powell et al. 2016). A similar complexity is seen in the role of financial incentives: while financial incentives may legitimately motivate people to participate, they can also become problematic when they become too dominant.

10 Authenticity is not widely accepted in bioethics as a condition for autonomous action or voluntariness, as it would be too demanding and, thus, make most actions non-autonomous or involuntary (Faden and Beauchamp 1989; Nelson et al. 2011). Although we do not have the space here to discuss this in full detail, we would like to point out that Christman’s test is hypothetical and requires the absence of alienation, not some type of reflective endorsement. This is a standard we think most people and most actions can make.

11 It is good to note that this does not imply that a participant that participates involuntarily because of the money is necessarily wronged or forced to participate by the trialist or recruiter who offered the money. People can do something involuntarily without being wronged by another person, even when another, identifiable person has caused you to act involuntarily. You can feel, for example, emotional pressure from a loved one to do something that you do not want to do, without that person wronging you. Emotional pressure may cause you to act against your will, but, when their emotions are real and not actively used to pressure you, it can be argued that your loved one has not wronged you.

12 We thank an anonymous reviewer for this suggestion.

13 These questions are not only important for the ethics of financial incentives, but also for the challenge to retain participants in a trial. Although there is more data needed to confirm this, it is plausible that people who are only motivated to participate in a trial by extrinsic factors—such as financial incentives—are less inclined to stay in a trial, especially when those extrinsic factors disappears—e.g., when the incentive has been paid. There may, thus, be a connection between the level of autonomy of a decision to participate and the decision to stay in a trial. If this is true, there are not only ethical reasons to keep financial incentives low, but also pragmatic reasons.
